# Hospitals by day, dispensaries by night: Hourly fluctuations of maternal mortality within Mexican health institutions, 2010–2014

**DOI:** 10.1371/journal.pone.0198275

**Published:** 2018-05-31

**Authors:** Hector Lamadrid-Figueroa, Alejandra Montoya, Jimena Fritz, Eduardo Ortiz-Panozo, Dolores González-Hernández, Leticia Suárez-López, Rafael Lozano

**Affiliations:** 1 National Institute of Public Health, Cuernavaca, Morelos, México; 2 Institute for Health Metrics and Evaluation, Seattle, WA, United States of America; University of Oxford, UNITED KINGDOM

## Abstract

**Background:**

Quality of obstetric care may not be constant within clinics and hospitals. Night shifts and weekends experience understaffing and other organizational hurdles in comparison with the weekday morning shifts, and this may influence the risk of maternal deaths.

**Objective:**

To analyze the hourly variation of maternal mortality within Mexican health institutions.

**Methods:**

We performed a cross-sectional multivariate analysis of 3,908 maternal deaths and 10,589,444 births that occurred within health facilities in Mexico during the 2010–2014 period, using data from the Health Information Systems of the Mexican Ministry of Health. We fitted negative binomial regression models with covariate adjustment to all data, as well as similar models by basic cause of death and by weekdays/weekends. The outcome was the Maternal Mortality Ratio (MMR), defined as the number of deaths occurred per 100,000 live births. Hour of day was the main predictor; covariates were day of the week, c-section, marginalization, age, education, and number of pregnancies.

**Results:**

Risk rises during early morning, reaching 52.5 deaths per 100,000 live births at 6:00 (95% UI: 46.3, 62.2). This is almost twice the lowest risk, which occurred at noon (27.1 deaths per 100,000 live births [95% U.I.: 23.0, 32.0]). Risk shows peaks coinciding with shift changes, at 07:00, and 14:00 and was significantly higher on weekends and holidays.

**Conclusions:**

Evidence suggests strong hourly fluctuations in the risk of maternal death with during early morning hours and around the afternoon shift change. These results may reflect institutional management problems that cause an uneven quality of obstetric care.

## Introduction

Maternal mortality has been studied since at least the mid-18th century, thanks to the existence of long time series of vital statistics in Sweden and the United Kingdom [1,2]]. In Mexico, despite counting on vital statistics of maternal deaths practically from the early 20th century, information was only consolidated beginning in 1940 [3]]. In 1940 a maternal death occurred every 2 hours or 13 per day, whereas by 2014 this decreased to one death every 8 hours or 3 per day. However, the trend in the number of births is opposite, thus the Maternal Mortality Ratio (MMR) fell 92.3% over this period. Between 1940 and 1980, the MMR decreased 6-fold falling from 536 to 95 deaths per 100,000 live births; in the following 20 years it halved falling from 94 to 47, and after the year 2000 the decline has been only 13% [3].

In terms of measurement, important advancements in the registration of maternal deaths have occurred in Mexico. After the implementation of the Intentional Search and Reclassification of Maternal Deaths System (BIRMM, acronym in Spanish) in 2002, approximately 20% maternal deaths originally classified as non-maternal deaths were recovered [4–6]. Due to improving registries, indirect maternal deaths have been analyzed with a greater degree of certainty [7], as well as the contribution of late maternal deaths and obstetric sequelae deaths to maternal mortality in Mexico [8]. Nevertheless, despite the gains in the measurement of maternal mortality, the MMR has not declined as expected, which points to insufficient knowledge of its determinants. Once regarded as one of the most important factors in explaining maternal deaths in Mexico: access to health services has dramatically improved. According to publicly available data from the Ministry of Health, 98% of all deliveries in Mexico in the 2010–2014 period occurred in a clinic or hospital. However, 83% of all maternal deaths occurred within these same facilities. Taken together, these numbers point to the problem of lingering maternal mortality being related to the quality of obstetric care within institutions.

This study seeks to use the good quality information on maternal mortality in Mexico to identify its determinants. Of particular interest is a seldom studied factor, which is the hour and day of the week on which death occurs as a function of variations in the continuum of the quality of maternal care within health facilities. Discussion on varying health outcomes as a function of the hour of day, due to understaffing and other problems outside the usual “office hours” has been going on for several years [9], with at least one study estimating an association between “out-of-hours” births and neonatal mortality in the UK.[10]. A larger number of studies have been conducted however, on daily variations in clinical outcomes, collectively called the “weekend effect”.[11–14] Evidence from the UK shows a weekend effect on perinatal mortality and obstetric practices, which begs the question on whether similar variations could occur during the course of a single day[15,16].

An old saying among Mexican health professionals states that hospitals in Mexico are “hospitals by morning, clinics by the afternoon, and dispensaries by night”; this adage is a reflection of widespread anecdotal observations about hourly fluctuations of the quality of care in Mexican hospitals, which if actually true, should cause risk variations within the course of a day. Consequently, we set out to explore the relationship between maternal deaths and the hour of day, to ascertain whether hourly risk fluctuations are consistent with what would be expected if quality of obstetric care does indeed vary in 24 hour cycles.

## Methods

The study was approved by the Ethics Committee of the National Institute of Public Health of Mexico (authorization number CI: 1340).

### Data sources

We obtained data on 5,232 pregnancy-related deaths and 10,781,915 births occurred during the 2010–2014 period from datasets of the BIRMM and the Ministry of Health Birth Information Subsystem (SINAC, acronym in Spanish), respectively. We retrieved information on the place and conditions of the death or birth, as well as sociodemographic characteristics and obstetric history of the mother. Additionally, we obtained information on the Marginalization Index of the mother’s place of residence, a measure defined at the municipality level that summarizes access to education, proper housing, income and remoteness of dwellings [17].

### Variable definitions

#### Births, maternal deaths and maternal mortality ratio

We retrieved information on the 4,363 pregnancy related deaths and 10,589,444 births that occurred within a medical facility in Mexico during the study period, thus excluding 16.6% of all pregnancy related deaths and 1.8% of births, which occurred at home, or in public places. As for MMR calculation purposes maternal deaths are those that occurred during pregnancy or within 42 days after the termination of pregnancy; we excluded 455 late maternal deaths (deaths from any obstetric cause occurring more than 42 days but less than one year after delivery; ICD-10 code O96) and deaths due to sequelae of obstetric causes (deaths from any direct obstetric cause occurring one year or more after delivery; ICD-10 code O97) from the analysis, thus yielding a final analytic sample of 3,908 deaths. The performance of a c-section was included as a covariate. The unadjusted or crude MMR was obtained dividing the number of deaths by the number of live births [18].

#### Time of day, community and facility level variables

The time of day in which deaths occurred was the main independent variable, and it was defined as integer numbers from 0 to 23. From the date of death or delivery, we obtained the day of the week and a dichotomous variable that indicated whether it corresponded to a working day (Monday to Friday) or a non-working day (Saturdays, Sundays or holidays). We considered the geographical region within Mexico defined as north, central, and south, following the proposal of the National Health and Nutrition Survey (ENSANUT, acronym in Spanish) 2012 [19]. The marginalization index of the municipality of residence was grouped it into three categories (high or very high, average, and low or very low) [17].

#### Individual level covariates

The mother’s age, years of schooling, type of health insurance, and number of previous pregnancies were included as covariates. Age was categorized into 10-year groups. Years of education was grouped into four categories (0–5, 6–8, 9–11 and 12 or more). The number of previous pregnancies was grouped into three categories (0, 1 and 2 or more).

#### Causes of death

Basic causes of death were grouped into nine mutually exclusive categories as defined by the ICD-10[18]: abortion; hypertensive disorders of pregnancy, delivery and *puerperium*; obstetric hemorrhage; pregnancy related infections; other obstetric complications; unforeseen treatment complications; non-obstetric complications, undetermined; other codes of interest and contributing conditions. The complete classification appears in the Appendix (S1 File).

### Statistical analysis

#### Data quality

In order to improve the completeness of the datasets we imputed missing and non-specified data using the Multiple Imputation by Chained Equations method (MICE) [20]. In order to evaluate the plausibility of the imputations, we compared their distribution to that of observed data [21]. The percentage of imputed values varied by covariate, ranging from 0.2% in the case of level of marginalization in the municipality of residence to 5.7% in the case of type of health insurance (S1 Table).

#### Merger of deaths and births datasets

Deaths and births information are stored in two separate datasets that cannot be merged by normal means at the individual level as there is a lack of an identifying variable. Thus, we collapsed individual level data was into covariate patterns (groupings of observations that share the same values of the covariates of interest), forming a multidimensional contingency table. Each cell of said table contains the number of deaths and births that occurred for women with that exact pattern of covariates during the study period.

#### Adjusting for the time-wise correlation between births and deaths

**Fig 1:**
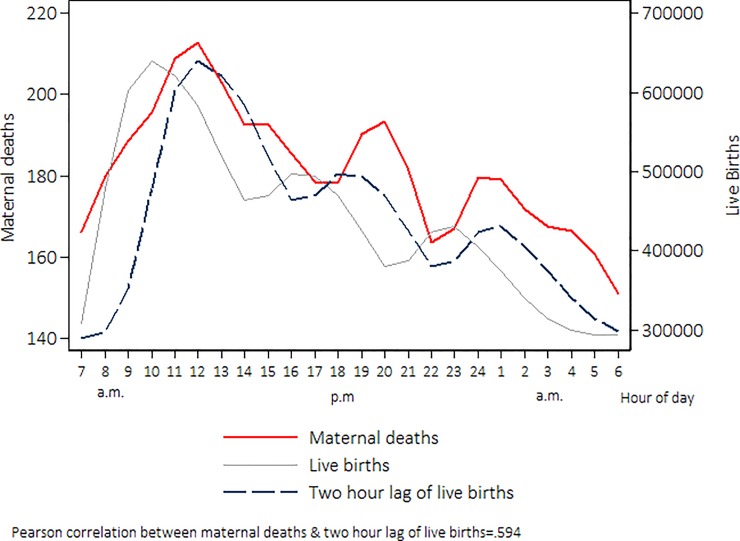
Distribution of births and deaths per hour of occurrence in health institutions, Mexico 2010–2014. Distributions were plotted with smoothing by weighted local regression (LOWESS), with a bandwidth of 0.2.

Since most of maternal deaths occurred nearly after the moment of birth, it is expected for the variability cycles of death as a function of time at any given day to closely match the analog cycles of births during the same day. However, peaks and valleys in the death and birth functions are not in harmony, as deaths do not tend to occur immediately at the moment of birth but rather some time after it; thus a simplistic analysis dividing the number of deaths occurred at any particular hour by the number of births occurred in the same lapse would likely produce biased risk estimates. Given this, we analyzed fluctuations of births as a function of time, introducing lags in the hour of birth (in 1 hour increments), thus displacing the birth function seeking to maximize its Pearson’s correlation to the death function, as long as the lag was not larger than 12 hours. The optimal estimated lag to harmonize the daily fluctuations of deaths and births was 2 hours, achieving a Pearson’s correlation of 0.68 between the functions (This means the typical maternal death in Mexican hospitals occurs 2 hours after delivery). The displacement and harmonization of both functions is apparent on Fig 1. Lags varied by cause of death, thus we adjusted each stratified model with the optimal lag that maximized the correlation between the birth and death functions; the complete lag analysis results appear in the Appendix (S1 File).

#### MMR estimation

We fitted negative binomial regression models to estimate covariate-adjusted MMR and 95% uncertainty intervals (95% U.I.). Uncertainty intervals are numerically and analytically equivalent to confidence intervals, however, since we analyzed census-type data of deaths and births, the standard errors do not convey their usual sense of “sampling” error, but rather the volatility of the parameters given by the variability in death counts.

The model specification was:

$$\log\left(\frac{MD_{p}}{B_{p}}\right)=\beta_{0}+\sum_{pj}\beta_{pj}X_{pj}+\varepsilon_{p}$$ (1)

Where *MD_p_* is the number of maternal deaths occurred within covariate pattern *p*; *B_p_* is the number of births (*exposure)* in the same covariate pattern; *X_pj_* represents a vector of the previously described explanatory variables and *β_pj_* is a vector of regression parameters.

For simplicity’s sake, we use the term “risk” as a synonymous of MMR in the results section. All the analyses were performed with Stata v 15.0 (StataCorp, College Station, TX). All estimates with a p<0.05 were considered to be statistically significant.

## Results

The characteristics of the study population appear in Table 1. The MMR within the Mexican health institutions was 36.9 maternal deaths per 100,000 live births during the 2010 to 2014 period, which was somewhat lower than the overall MMR (43.8 deaths per 100,000 live births).

**Table 1:** Descriptive statistics of study population, Mexico 2010–2014.

		Deaths		Births	
	n =	3,908		10,589,444	
		Freq.	%	Freq.	%
**Women Characteristics**					
Age					
	10–19	548	14	2,174,596	20.5
	20–29	1,622	41.5	5,724,019	54.1
	30–39	1,521	38.9	2,496,185	23.6
	40 ó +	217	5.6	194,644	1.8
Years of educational attainment					
	0–5 years	740	18.9	831,823	7.9
	6–8 years	988	25.3	2,110,087	19.9
	9–11 years	1,140	29.2	4,162,268	39.3
	12 years or more	1,040	26.6	3,485,266	32.9
Type of health insurance					
	None	2,878	73.6	7,264,196	68.6
	Private workers	787	20.1	2,741,679	25.9
	State workers	243	6.2	583,569	5.5
Number of previous pregnancies					
	1 or more	2,571	65.8	6,629,638	62.6
	None	1,337	34.2	3,959,806	37.4
Region of Residence					
	North	807	20.6	2,503,711	23.6
	Center	1,716	43.9	5,055,682	47.7
	South	1,385	35.4	3,030,051	28.6
Level of marginalization in the municipality of residence					
	Higher	702	18	968,102	9.1
	Medium	805	20.6	2,081,297	19.7
	Low	2,401	61.4	7,540,045	71.2
**Characteristics of deaths and births**				** **	** **
Type of childbirth					
	Vaginal Birth /other	2,362	60.4	5,646,903	53.3
	C-Section	1,503	38.5	4,925,625	46.5
Place of occurrence					
	Public Institution^2^	3,531	90.4	8,422,906	79.5
	Private Institution	377	9.6	2,166,538	20.5
Day of the week					
	Monday-Friday	2,751	70.4	7,837,318	74.0
	Weekends and Holidays^3^	1,157	29.6	2,944,597	27.8
Hour of day					
	Morning	1,198	30.7	3,950,879	37.3
	Afternoon	1,185	30.3	3,579,075	33.8
	Night	1,525	39.0	3,059,490	28.9

The lowest MMR occurred during the morning shift, especially between 09:00 am and 13:00, and the highest occurred during the night shift, with the afternoon shift in an intermediate position. Risk was significantly higher during early morning hours: 44.2 (95% U.I. 41.1, 47.6) deaths per 100,000 live births between 00:00 am and 08:00 am; this was 63% higher than the lowest MMR during the day, occurring at 12:00 (27.1 deaths per 100,000 live births [95% U.I.: 23.0, 32.0]) and 55% greater than the risk occurring between 09:00 and 12:00 (29.1 deaths per 100,000 live births, 95% U.I.: 26.6, 31.9). The highest risk occurred at 6 am (52.5, 95% U.I.: 46.3, 62.2), followed by 5 am (52.4, 95% UI: 46.2, 64.8) and 7 am (51.6, 95% U.I.: 43.5, 61.2).

**Fig 2:**
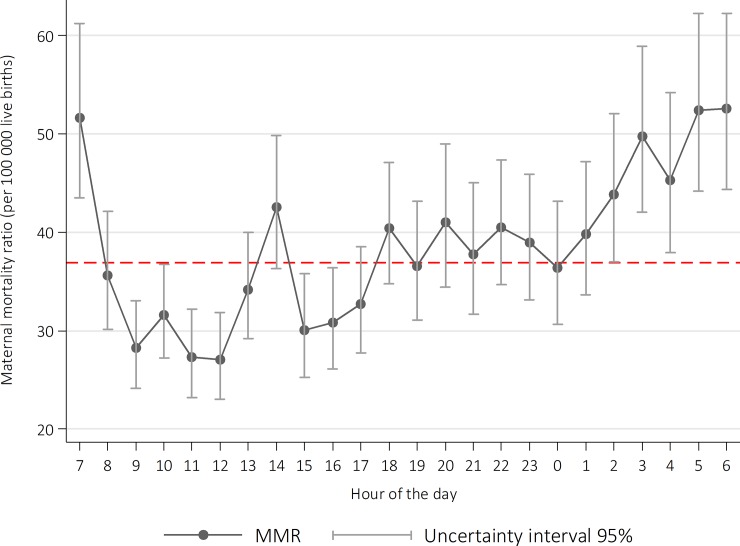
Maternal mortality ratio occurred in health care institutions, per hour of the day, Mexico 2010–2014. MMR estimated from negative binomial regression models, including two-hour lag for the time of death.

We observed that the MMR shows peaks (local maxima) that roughly coincide with shift change hours: that at 06:00–07:00, and another one at 14:00 (MMR = 42.5, which is 25% higher than the MMR at 13:00, and 41% higher than that at 15:00). We also observe a crescent linear trend from 00:00 culminating at 07:00 (Fig 2). The complete model estimates are shown in supplemental S2 Table.

**Fig 3:**
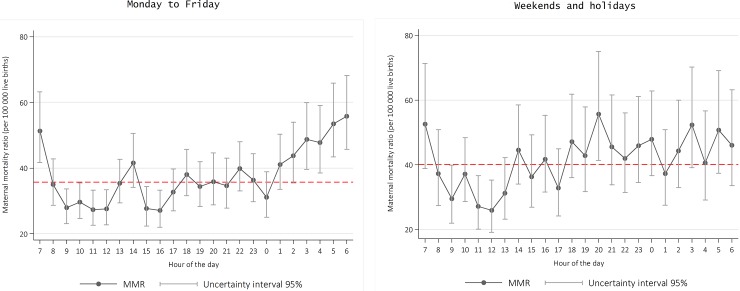
Maternal mortality ratio occurred in health care institutions, per hour of the day on working days and non-working days (weekend and holidays), Mexico 2010–2014. MMR estimated from negative binomial regression models, including two-hour lag for the time of death.

Daily risk fluctuations were much sharper from Mondays to Fridays; during weekends we did not observe such large fluctuations, but it is noteworthy that the overall MMR during holidays was significantly larger than that of weekdays (40.1 vs. 35.7, respectively, p = 0.02). We also observed that the MMR during weekends and holidays can be classified in two large blocks: it tends to remain below or near the mean from 08:00 to 17:00, and above the mean from 17:00 to 07:00 the next morning (Fig 3).

**Fig 4:**
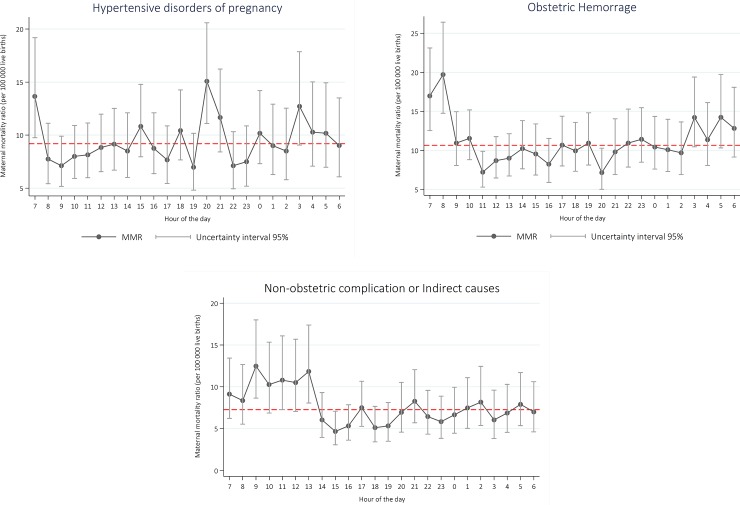
Maternal mortality ratio occurred in health care institutions, per hour of the day and cause of death, Mexico 2010–2014. MMR estimated from negative binomial regression models, including two-hour lag for the time of death.

Analyzing hourly risk by specific maternal causes, we observed fluctuations in hemorrhage mortality, with a 2-fold wide gap between the lowest risk, occurring at 10:00 (5.3 deaths due to hemorrhage per 100,000 live births) and the highest (11.5 deaths due to hemorrhage per 100,000 live births), occurring at 6:00. In the case of deaths due to hypertensive disorders, a much sharper peak occurred at 20:00 and another one at 7:00 (12.9 deaths per 100,000 live births). Deaths by indirect causes followed the same general pattern with an elevated risk between 7:00 and 8:00 that, in the latter case, was 2.65 times higher than the risk observed at 11:00 (Fig 4).

Finally, with the above results we proposed two hypothetical scenarios aiming to advance the concept of avoidable deaths. Data showed that there was a daily time span with much lower mortality between 9:00 and 12:00. In an ideal scenario in which the MMR could uniformly be lowered to levels experienced in Mexican institutions between 9:00 and 12:00 (29.1 deaths per 100,000 live births), 834 deaths could have been avoided during the study period (95% U.I.: 538, 1098). Moreover, if the MMR could be lowered to the lowest observed (at 12:00: 27.1 deaths per 100,000 live births), 1045 deaths (95% U.I.: 527, 1478), could have been averted during the period, corresponding to 38% of all maternal deaths occurred within Mexican health facilities between 2010 and 2014.

## Discussion

### Main findings

We confirmed an increased risk during the night shift, particularly during early morning hours; regardless of the cause of death. Additionally a weekend effect on maternal mortality was evident, as well as a particular pattern of excess maternal mortality around shift changes. If the institutional MMR in Mexico would have uniformly been at its minimum value of 27.1, which occurred at noon, the overall country MMR during the 2010–2014 period (excluding late and sequelae-related maternal deaths) would have been 34.1 deaths per 100,000 live births; 22% less than the observed MMR of 44 deaths per 100,000 live births.

### Strengths and limitations

We offer a systemic analysis of the MMR, in spite of the limitations of the available data, presenting a simple way to incorporate factors related to access and quality of health services into the analyses, contributing to the study of organizational determinants of maternal deaths. Moreover, conclusions are supported from analyzing data from a vast majority of deaths and births (close to a census) occurred in the country’s hospitals and clinics during the study period, and are thus mostly free of sampling error.

The most important limitation of the analysis is that the construction of the MMR is not defined at the individual woman, as we are linking deaths and births based solely on the timing of their occurrence, constructing a dataset where observations correspond to covariate patterns and not to individual women; with the drawback that individual level predictions are impossible. It would be a far better approach to identify the outcome of each particular pregnancy in terms of eventual death or survival; however limitations of the data make it impossible.

Due to limitations of the data, some confounding may be present as women that seek attention during night hours may be different than those who are cared for in the morning hours, which include women programmed for elective cesarean section or delivery induction; women seeking care at night may inherently have an urgency (and higher risk) as otherwise they might decide to wait until the morning to seek attention. The ability to adjust for the performance of a c-section, accounts for potential confounding due to elective cesareans (theoretically low risk) being performed mostly during the morning shift. Some residual confounding may persist however because of our inability to distinguish between elective and emergency procedures. It is important to note, however, that night-day patient differences could not account for the risk peak occurring around the afternoon shift.

Another important limitation is that in order to establish causal relationships of death with factors such as understaffing, the relevant time would not be that of death itself but rather that of the critical event or events that led to death (which are of course the most important in terms of future prevention). The problem is that these may have occurred hours or even days before. For example: a critical event such as a stroke due to a poorly treated eclampsia could occur around 9 AM with death following at 9 PM; if this happens we could be wrongly attributing death to events occurring at night which is clearly not the case. If one views hour of death as a proxy of the hour of the critical event that led to it, then the resulting misclassification of the outcome variable could indeed bias the results in a way that is hard to predict and increase the uncertainty in the estimates; we believe however that bias is attenuated by the lag introduced in the analyses.

Finally our analysis is not inclusive of 17% of deaths and 2% of births that occurred outside health facilities during the study period. Although this might seem as a limitation, we believe adding them to the analysis would obscure the patterns made evident by our work. Time patterns within the health system reflect mostly on issues of uneven quality of care, whereas deaths occurred outside the system almost surely follow different time patterns that respond to different determinants.

### Interpretation

In Mexico, maternal mortality has indeed decreased, but not to the extent that was hoped for [22][23]. Few studies on maternal mortality determinants have tried to adjust for confounders or to incorporate analytical variables related to the organization of health services provision; this study does both and shows results of the hourly excess maternal deaths within Mexican hospitals.

The study of the effect of hour and day of the week on the quality and outcomes of health services seeks to ascertain whether outcomes such as mortality, prolonged stays, complications, etc. are related to the time on which services were provided. Palmer et al [16] studied 1.3 million obstetric events in United Kingdom hospitals during 2015, finding 7% greater odds of perinatal deaths in weekend admissions compared to weekday admissions. These same authors stated that should this “weekend effect” be eliminated, up to 770 perinatal deaths would have been avoided during the studied period. Some studies in other hospital areas showed no weekend effect on mortality [24], although most studies do show an increased risk of dying related to weekend admissions. When the shift or hour of admission has been analyzed, results have shown an excess risk for patients during the night shift [25], which could be explained by understaffing and a lack of equipment or consumables needed to solve emergencies. A study of hospital discharge records of the USA showed excess mortality on hospitals with a small number of doctors and nurses [26]. Shulkin (2008) discussed how quality of health care varies between shifts, with night shifts and weekends being the worst performers possibly due to the absence of directive personnel, and overall scarcity of staff [8]. In Mexico, there appears to be a wide gap between shifts at different hours of day. To put numbers in context, the lowest MMR estimated in our study of 27.1 at noon is comparable to that of the United States (2016 estimates), while the highest, at 52.5, is comparable to that of Iraq or the Maldives.[27] This variation is worrisome as patients need constant quality care 24 hours a day, consequences of the opposite being higher mortality and readmissions rates, more surgical complications and more possibilities of medical errors.

Recent studies in the UK, specifically tested for whether a 24-hour presence of an obstetrician in the labour ward, resulted in improved outcomes for women and newborn [28,29] Although no significant association was found, the situation in Mexico around this specific matter may not be comparable for a variety of reasons. Firstly, maternal and neonatal mortality rates in Mexico are much higher than in the UK (maternal mortality was estimated to be almost seven times as high and neonatal mortality almost three times as high in 2016)[30]. Secondly, factors such as lack of antenatal care are quite common in Mexico and bear heavily in the outcome of the pregnancy, thus making the presence of experienced staff at the moment of delivery much more crucial as complications are likelier to arise [31]. In the UK, most low-risk deliveries are attended by experienced professional midwives. Midwives are very scarce in Mexico and many, if not most, institutional deliveries are attended by Medical Interns (5^th^ year medical students), whom frequently lack the experience or knowledge to properly identify or manage emerging problems during delivery; at least one study in Mexico has documented an important lack of use for evidence based-practices inside health facilities [32]. This makes the presence of an obstetrician in the labour ward much more relevant. Lastly, understaffing is not limited to obstetricians, but also to other key personnel such as anesthesiologists and nurses, which causes a delay in case of the need for an emergency cesarean section, for instance.

Finally, we could not locate literature documenting excess risk around shift changes, which are characterized by an exchange of information between the departing and arriving personnel in any hospital area. Shift change handovers comprise particular technical, ethical and organizational culture aspects that have been seldom studied, but have been recognized as difficult and probably important determinants of the quality of care [26].

## Conclusions

The findings of this study do not allow us to reach direct conclusions regarding the individual risk of dying due to maternal causes within Mexican hospitals and clinics as causal inferences are impossible due to the limitations of secondary data analyses. Rather, these results allow us to advance new hypotheses about the maternal mortality phenomenon, which are presumably relevant for most countries. More in-depth studies that collect first-hand data are needed to further understand the factors behind the excess mortality that occurs around shift changes, as well as that during night shifts and weekends. Accounting and adjusting for individual patient differences in the nocturnal vs. diurnal shifts is crucial if causal relations between factors such as understaffing and mortality risk are to be confirmed. Regardless of future research on the matter, the implementation of routine Maternal Deaths Surveillance and Response efforts is crucial to fully delineate the critical events that lead to death in each particular case [32], thus allowing for eventually successful preventive actions.

The evidence made available by our study, if eventually confirmed, calls for new ways to improve the organization of obstetric care services and highlights the importance of implementing health policies that foster full staffing during night shifts and weekends. In the United Kingdom, for example, after introduction of an enhanced, consultant-led weekend service in acute medicine programme, in-hospital mortality was significantly reduced [33]. Another possible option would be pay per performance; hospitals also need to publicize their out-of-hours improvements in a consumer-friendly way to help patients make informed decisions (http://patientcarelink.org), to achieve a competitive environment. Hospital administrators should also be aware of the situations that hospitals face, take into account the views and needs of staff, and pay more attention to the needs of patients and their families [9]. All this would improve quality of care thus avoiding unnecessary deaths.

It is important to consider that Mexico fell short of reaching the Millenium Development Goal No. 5 for the country and is having problems dealing with Sustainable Development Goal 3.1. To reach these commitments, we have to look for maternal deaths that can potentially be avoided, and one way to do that is by reaching a better, and constant, quality of care. We need to move towards increasing the availability of health providers at all times, not only with residents and students, but rather with fully staffed hospitals and clinics in order to guarantee the best possible health care 24 hours a day, 7 days a week.

## Supporting information

S1 Table Percentage of missing values imputed in the births and deaths datasets. (DOCX)

S2 Table Negative binomial regression model results. (DOCX)

S1 File Appendix. Complete maternal death classification; hourly c-section percentages; complete lag analysis results.
(DOCX)
