# Supplementary material for: Hospitals by day, dispensaries by night: Hourly fluctuations of maternal mortality within Mexican health institutions, 2010–2014
Source: PLoS One. 2018 May 31;13(5):e0198275. doi: 10.1371/journal.pone.0198275 (PMC5979009; doi:10.1371/journal.pone.0198275)
Supplement: S1 Table — (DOCX) [file pone.0198275.s001.docx]

Table A1. Percentage of missing values imputed

| Variables | missing in maternal deaths database | missing in births database |
| --- | --- | --- |
| Age | 0.0% | 0.4% |
| Years of educational attainment | 5.8% | 1.5% |
| Type of health insurance | 6.1% | 3.1% |
| Gravidity | 0.0% | 0.0% |
| Region of Residence | 0.0% | 0.0% |
| Level of marginalization in the municipality of residence | 0.1% | 0.7% |
| Place of attention | 1.1% | 0.0% |
| Day of the week | 0.0% | 0.1% |
| Hour of day | 0.7% | 0.0% |
