# Supplementary material for: Hospitals by day, dispensaries by night: Hourly fluctuations of maternal mortality within Mexican health institutions, 2010–2014
Source: PLoS One. 2018 May 31;13(5):e0198275. doi: 10.1371/journal.pone.0198275 (PMC5979009; doi:10.1371/journal.pone.0198275)
Supplement: S2 Table — (DOCX) [file pone.0198275.s002.docx]

Table A2. Complete Negative binomial regression model results.

|  |  | **General model** | | **Monday-Friday** | | **Weekends** | | **Hypertensive disorders in pregnancy, childbirth, and the puerperium** | | **Obstetric hemorrhage** | | **Non-obstetric complications or Indirect causes** | |
| --- | --- | --- | --- | --- | --- | --- | --- | --- | --- | --- | --- | --- | --- |
| Variables | | IRR | UI 95% | IRR | UI 95% | IRR | UI 95% | IRR | UI 95% | IRR | UI 95% | IRR | UI 95% |
|  |  |  |  |  |  |  |  |  |  |  |  |  |  |
|  |  |  |  |  |  |  |  |  |  |  |  |  |  |
| Age |  |  |  |  |  |  |  |  |  |  |  |  |  |
|  | 10-19 | - |  | - |  | - |  | - |  | - |  | - |  |
|  | 20-29 | 1.556*** | (1.405 - 1.723) | 1.595*** | (1.410 - 1.805) | 1.476*** | (1.230 - 1.771) | 1.686*** | (1.357 - 2.095) | 1.591*** | (1.190 - 2.128) | 1.268** | (1.057 - 1.522) |
|  | 30-39 | 3.634*** | (3.251 - 4.062) | 3.621*** | (3.167 - 4.141) | 3.684*** | (3.017 - 4.498) | 4.519*** | (3.579 - 5.706) | 4.627*** | (3.428 - 6.244) | 2.520*** | (2.051 - 3.096) |
|  | 40 or more | 6.448*** | (5.490 - 7.572) | 6.091*** | (5.015 - 7.398) | 7.457*** | (5.602 - 9.927) | 9.034*** | (6.571 - 12.42) | 6.719*** | (4.494 - 10.05) | 3.761*** | (2.677 - 5.282) |
| Years of educational attainment | | | |  |  |  |  |  |  |  |  |  |  |
|  | 0-5 years | - |  | - |  | - |  | - |  | - |  | - |  |
|  | 6-8 years | 0.682*** | (0.619 - 0.751) | 0.671*** | (0.598 - 0.753) | 0.706*** | (0.590 - 0.845) | 0.722*** | (0.591 - 0.881) | 0.805** | (0.648 - 0.999) | 0.599*** | (0.496 - 0.722) |
|  | 9-11 years | 0.449*** | (0.408 - 0.495) | 0.427*** | (0.380 - 0.479) | 0.508*** | (0.425 - 0.607) | 0.491*** | (0.402 - 0.601) | 0.502*** | (0.400 - 0.629) | 0.378*** | (0.313 - 0.456) |
|  | 12 years or more | 0.460*** | (0.414 - 0.512) | 0.445*** | (0.393 - 0.505) | 0.498*** | (0.410 - 0.606) | 0.508*** | (0.408 - 0.632) | 0.555*** | (0.433 - 0.711) | 0.457*** | (0.374 - 0.558) |
| Type of health insurance | | |  |  |  |  |  |  |  |  |  |  |  |
|  | Non (ref) |  |  |  |  |  |  |  |  |  |  |  |  |
|  | Private workers | 0.363*** | (0.286 - 0.461) | 0.381*** | (0.287 - 0.505) | 0.323*** | (0.208 - 0.503) | 0.0977*** | (0.0609 - 0.157) | 0.156*** | (0.0890 - 0.275) | 0.418*** | (0.259 - 0.674) |
|  | State workers | 1.067 | (0.891 - 1.276) | 1.120 | (0.909 - 1.379) | 0.936 | (0.657 - 1.334) | 0.920 | (0.623 - 1.358) | 0.831 | (0.547 - 1.260) | 1.045 | (0.717 - 1.523) |
| Number of previous pregnancies | | | |  |  |  |  |  |  |  |  |  |  |
|  | 1 or more | - |  | - |  | - |  | - |  | - |  | - |  |
|  | Primiparous | 1.600*** | (1.485 - 1.724) | 1.550*** | (1.417 - 1.695) | 1.707*** | (1.491 - 1.955) | 1.834*** | (1.577 - 2.134) | 0.709*** | (0.576 - 0.871) | 1.667*** | (1.445 - 1.923) |
| Region of mother residence | | |  |  |  |  |  |  |  |  |  |  |  |
|  | North (ref) | - |  | - |  | - |  | - |  | - |  | - |  |
|  | Center | 1.024 | (0.942 - 1.114) | 1.016 | (0.919 - 1.123) | 1.043 | (0.893 - 1.218) | 1.030 | (0.865 - 1.227) | 1.064 | (0.865 - 1.309) | 0.862* | (0.738 - 1.006) |
|  | South | 1.052 | (0.956 - 1.157) | 1.023 | (0.912 - 1.147) | 1.122 | (0.942 - 1.336) | 0.987 | (0.808 - 1.206) | 1.195 | (0.949 - 1.505) | 0.981 | (0.821 - 1.171) |
| Level of marginalization^1^ in the municipality of residence | | | | |  |  |  |  |  |  |  |  |  |
|  | Higher (ref) | - |  | - |  | - |  | - |  | - |  | - |  |
|  | Medium | 0.683*** | (0.615 - 0.759) | 0.677*** | (0.596 - 0.768) | 0.699*** | (0.578 - 0.845) | 0.530*** | (0.430 - 0.653) | 0.586*** | (0.467 - 0.736) | 0.821* | (0.664 - 1.015) |
|  | Low | 0.683*** | (0.617 - 0.756) | 0.689*** | (0.610 - 0.779) | 0.671*** | (0.558 - 0.806) | 0.523*** | (0.428 - 0.640) | 0.511*** | (0.407 - 0.640) | 0.830* | (0.676 - 1.020) |
| Type of childbirth | |  |  |  |  |  |  |  |  |  |  |  |  |
|  | Vaginal Birth (ref) | - |  | - |  | - |  | - |  | - |  | - |  |
|  | C-Section | 0.863*** | (0.806 - 0.924) | 0.836*** | (0.771 - 0.908) | 0.940 | (0.829 - 1.065) | 2.202*** | (1.919 - 2.527) | 1.015 | (0.865 - 1.191) | 0.714*** | (0.624 - 0.817) |
| Place of occurrence | |  |  |  |  |  |  |  |  |  |  |  |  |
|  | IMSS (ref) | - |  | - |  | - |  | - |  | - |  | - |  |
|  | ISSSTE | 2.536*** | (1.989 - 3.233) | 2.348*** | (1.759 - 3.133) | 3.007*** | (1.925 - 4.697) | 6.717*** | (4.253 - 10.61) | 4.901*** | (2.793 - 8.599) | 2.719*** | (1.678 - 4.407) |
|  | SSA | 1.310** | (1.019 - 1.685) | 0.986 | (0.724 - 1.343) | 2.438*** | (1.559 - 3.813) | 1.189 | (0.714 - 1.983) | 1.419 | (0.776 - 2.595) | 0.960 | (0.548 - 1.682) |
|  | Other Public ^3^ | 0.896* | (0.799 - 1.005) | 0.862** | (0.751 - 0.989) | 0.978 | (0.794 - 1.205) | 0.759** | (0.594 - 0.969) | 1.070 | (0.833 - 1.375) | 0.824 | (0.651 - 1.043) |
|  | Private | 0.451*** | (0.400 - 0.507) | 0.449*** | (0.390 - 0.516) | 0.447*** | (0.358 - 0.558) | 0.190*** | (0.143 - 0.253) | 0.790* | (0.623 - 1.001) | 0.374*** | (0.290 - 0.482) |
| Day of the week | |  |  |  |  |  |  |  |  |  |  |  |  |
|  | Monday-Friday (ref) | - |  | - |  | - |  | - |  | - |  | - |  |
|  | Weekends and Holidays^3^ | 1.100*** | (1.028 - 1.177) |  |  |  |  | 1.125* | (0.978 - 1.295) | 0.999 | (0.850 - 1.175) | 1.084 | (0.952 - 1.235) |
| Hour of day | |  |  |  |  |  |  |  |  |  |  |  |  |
|  | 7 (ref) | - |  | - |  | - |  | - |  | - |  | - |  |
|  | 8 | 0.737*** | (0.596 - 0.913) | 0.704*** | (0.546 - 0.907) | 0.824 | (0.555 - 1.224) | 0.555** | (0.354 - 0.869) | 0.915 | (0.550 - 1.522) | 1.249 | (0.852 - 1.830) |
|  | 9 | 0.547*** | (0.444 - 0.676) | 0.532*** | (0.415 - 0.681) | 0.592*** | (0.397 - 0.881) | 0.396*** | (0.259 - 0.607) | 1.362 | (0.849 - 2.185) | 0.772 | (0.519 - 1.148) |
|  | 10 | 0.581*** | (0.473 - 0.714) | 0.542*** | (0.423 - 0.693) | 0.688* | (0.472 - 1.002) | 0.438*** | (0.290 - 0.661) | 1.121 | (0.680 - 1.851) | 0.810 | (0.562 - 1.168) |
|  | 11 | 0.531*** | (0.429 - 0.657) | 0.506*** | (0.393 - 0.652) | 0.589*** | (0.399 - 0.869) | 0.431*** | (0.284 - 0.654) | 1.183 | (0.720 - 1.943) | 0.484*** | (0.322 - 0.726) |
|  | 12 | 0.490*** | (0.395 - 0.608) | 0.481*** | (0.373 - 0.621) | 0.507*** | (0.338 - 0.759) | 0.455*** | (0.302 - 0.685) | 1.160 | (0.706 - 1.906) | 0.575*** | (0.387 - 0.852) |
|  | 13 | 0.606*** | (0.490 - 0.750) | 0.626*** | (0.488 - 0.804) | 0.548*** | (0.365 - 0.822) | 0.484*** | (0.319 - 0.736) | 1.342 | (0.830 - 2.170) | 0.589*** | (0.399 - 0.871) |
|  | 14 | 0.766** | (0.619 - 0.947) | 0.759** | (0.587 - 0.980) | 0.776 | (0.528 - 1.141) | 0.523*** | (0.334 - 0.817) | 0.704 | (0.423 - 1.174) | 0.676** | (0.458 - 1.000) |
|  | 15 | 0.574*** | (0.460 - 0.717) | 0.517*** | (0.396 - 0.676) | 0.723 | (0.487 - 1.075) | 0.603** | (0.399 - 0.912) | 0.534** | (0.324 - 0.880) | 0.623** | (0.407 - 0.951) |
|  | 16 | 0.567*** | (0.457 - 0.704) | 0.491*** | (0.378 - 0.639) | 0.785 | (0.534 - 1.152) | 0.434*** | (0.283 - 0.665) | 0.606** | (0.376 - 0.977) | 0.532*** | (0.347 - 0.815) |
|  | 17 | 0.578*** | (0.465 - 0.719) | 0.562*** | (0.434 - 0.727) | 0.619** | (0.412 - 0.929) | 0.382*** | (0.244 - 0.598) | 0.845 | (0.537 - 1.329) | 0.698* | (0.473 - 1.029) |
|  | 18 | 0.690*** | (0.559 - 0.852) | 0.631*** | (0.491 - 0.813) | 0.850 | (0.580 - 1.244) | 0.513*** | (0.338 - 0.780) | 0.574** | (0.350 - 0.941) | 0.637** | (0.426 - 0.953) |
|  | 19 | 0.660*** | (0.532 - 0.819) | 0.602*** | (0.466 - 0.779) | 0.821 | (0.552 - 1.220) | 0.344*** | (0.214 - 0.553) | 0.599** | (0.360 - 0.996) | 0.698* | (0.470 - 1.035) |
|  | 20 | 0.722*** | (0.576 - 0.905) | 0.622*** | (0.473 - 0.819) | 0.997 | (0.670 - 1.485) | 0.920 | (0.610 - 1.388) | 0.787 | (0.476 - 1.303) | 0.442*** | (0.281 - 0.696) |
|  | 21 | 0.709*** | (0.568 - 0.886) | 0.652*** | (0.499 - 0.853) | 0.842 | (0.565 - 1.254) | 0.703 | (0.458 - 1.077) | 0.932 | (0.582 - 1.492) | 0.615** | (0.394 - 0.960) |
|  | 22 | 0.671*** | (0.542 - 0.831) | 0.646*** | (0.501 - 0.833) | 0.722 | (0.485 - 1.075) | 0.352*** | (0.220 - 0.563) | 0.720 | (0.442 - 1.172) | 0.691* | (0.453 - 1.052) |
|  | 23 | 0.670*** | (0.539 - 0.831) | 0.611*** | (0.471 - 0.793) | 0.821 | (0.556 - 1.213) | 0.379*** | (0.237 - 0.606) | 0.649* | (0.391 - 1.079) | 0.722 | (0.487 - 1.072) |
|  | 24 | 0.610*** | (0.488 - 0.762) | 0.520*** | (0.394 - 0.685) | 0.816 | (0.556 - 1.197) | 0.530*** | (0.343 - 0.820) | 0.737 | (0.449 - 1.209) | 0.639** | (0.424 - 0.964) |
|  | 1 | 0.713*** | (0.574 - 0.887) | 0.736** | (0.568 - 0.953) | 0.656** | (0.436 - 0.988) | 0.473*** | (0.298 - 0.750) | 0.826 | (0.506 - 1.351) | 0.606** | (0.398 - 0.920) |
|  | 2 | 0.730*** | (0.584 - 0.912) | 0.716** | (0.549 - 0.936) | 0.759 | (0.506 - 1.137) | 0.465*** | (0.287 - 0.752) | 0.919 | (0.553 - 1.527) | 0.565*** | (0.366 - 0.872) |
|  | 3 | 0.822* | (0.659 - 1.025) | 0.798* | (0.612 - 1.041) | 0.874 | (0.586 - 1.303) | 0.733 | (0.472 - 1.137) | 0.679 | (0.394 - 1.169) | 0.827 | (0.553 - 1.236) |
|  | 4 | 0.763** | (0.608 - 0.957) | 0.781* | (0.596 - 1.022) | 0.723 | (0.473 - 1.104) | 0.598** | (0.373 - 0.958) | 0.759 | (0.461 - 1.249) | 0.644** | (0.416 - 0.997) |
|  | 5 | 0.893 | (0.716 - 1.113) | 0.887 | (0.681 - 1.156) | 0.907 | (0.606 - 1.357) | 0.601** | (0.373 - 0.968) | 0.872 | (0.536 - 1.420) | 0.810 | (0.534 - 1.227) |
|  | 6 | 0.957 | (0.770 - 1.188) | 1.015 | (0.786 - 1.311) | 0.824 | (0.546 - 1.242) | 0.544** | (0.332 - 0.890) | 0.771 | (0.464 - 1.281) | 0.728 | (0.473 - 1.119) |
|  |  |  |  |  |  |  |  |  |  |  |  |  |  |

*** p<0.01, ** p<0.05, * p<0.1. IRR: Incidence Rate Ratio
