# Supplementary material for: Hospitals by day, dispensaries by night: Hourly fluctuations of maternal mortality within Mexican health institutions, 2010–2014
Source: PLoS One. 2018 May 31;13(5):e0198275. doi: 10.1371/journal.pone.0198275 (PMC5979009; doi:10.1371/journal.pone.0198275)
Supplement: S1 File — Complete maternal death classification; hourly c-section percentages; complete lag analysis results. (DOCX) [file pone.0198275.s003.docx]

**Appendix**

| The WHO Application of ICD-10 to deaths during pregnancy, childbirth and the puerperium: ICD-MM **CIE-MM** | | |
| --- | --- | --- |
| Groups of underlying causes of death during pregnancy, childbirth and the puerperium in mutually exclusive, totally inclusive groups [**3**](#_bookmark2) | | |
| Type | Group name/number | EXAMPLES of potential causes of death |
| Maternal death: direct | 1. Pregnancies with abortive outcome | Abortion, miscarriage, ectopic pregnancy and other conditions leading to maternal death and a pregnancy with abortive outcome |
| Maternal death: direct | 2. Hypertensive disorders in pregnancy, childbirth, and puerperium. | Edema, proteinuria and hypertensive disorders in pregnancy, childbirth and puerperium. |
| Maternal death: direct | 3. Obstetric hemorrhage | Obstetric diseases or conditions directly associated with hemorrhage |
| Maternal death: direct | 4. Pregnancy-related infection | Pregnancy-related, infection-based diseases or conditions |
| Maternal death: direct | 5. Other obstetric complications obstétricas | All other direct obstetric conditions not included in groups to 1–4 |
| Maternal death: direct | 6. Unanticipated complications of management | Severe adverse effects and other unanticipated complications of medical and surgical care during pregnancy, childbirth or puerperium. |
| Maternal death: indirect | 7. Non-obstetric complications | Non-obstetric conditions  • Cardiac disease (including pre-existing hypertension)  • Endocrine conditions  • Gastrointestinal tract conditions  • Central nervous system conditions  • Respiratory conditions  • Genitourinary conditions  • Autoimmune disorders  • Skeletal diseases  • Psychiatric disorders  • Neoplasms  • Infections that are not a direct result of   - pregnancy - Infecciones que no derivan directamente del embarazo |
| Maternal death: unspecified | 8. Unknown/undetermined | Maternal death during pregnancy, childbirth and the puerperium where the underlying cause is  unknown or was not determined |
| Death during pregnancy, childbirth and the puerperium | 9. Coincidental causes | Death during pregnancy, childbirth and the puerperium due to external causes |

**Distribution of hourly percentages of cesarean sections**

| Hour of the day | % |
| --- | --- |
| 7 | **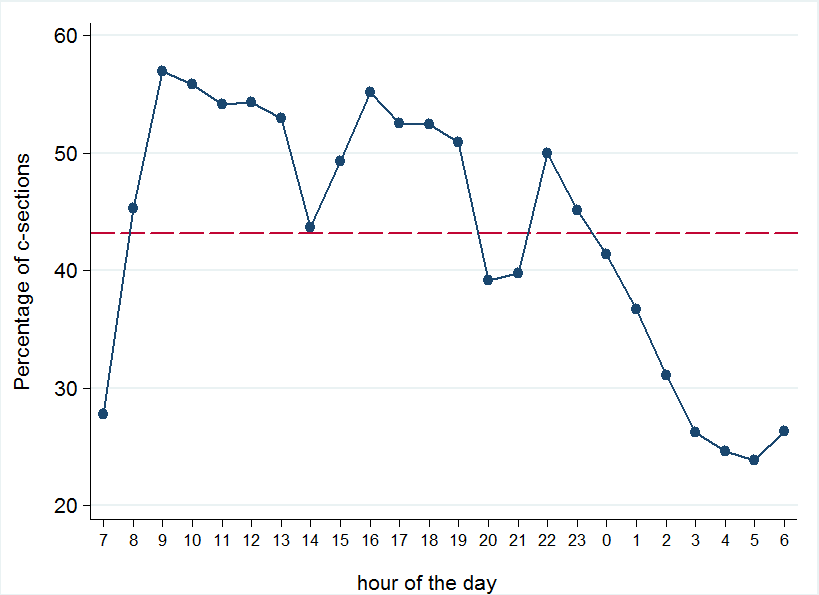**27.8% |
| 8 | 45.2% |
| 9 | 56.9% |
| 10 | 55.8% |
| 11 | 54.1% |
| 12 | 54.3% |
| 13 | 53.0% |
| 14 | 43.6% |
| 15 | 49.3% |
| 16 | 55.1% |
| 17 | 52.5% |
| 18 | 52.4% |
| 19 | 50.9% |
| 20 | 39.2% |
| 21 | 39.8% |
| 22 | 49.9% |
| 23 | 45.1% |
| 0 | 41.4% |
| 1 | 36.7% |
| 2 | 31.1% |
| 3 | 26.2% |
| 4 | 24.6% |
| 5 | 23.8% |
| 6 | 26.4% |

**Distribution of births and maternal deaths**

|  | **Raw distributions** | **Correlation between time of births and time of death with lags from 0 to 23 hours** | **Distributions with adjustment for optimal lag between time of birth and time of death** |
| --- | --- | --- | --- |
| **Births and maternal deaths occurred inside the health system** | 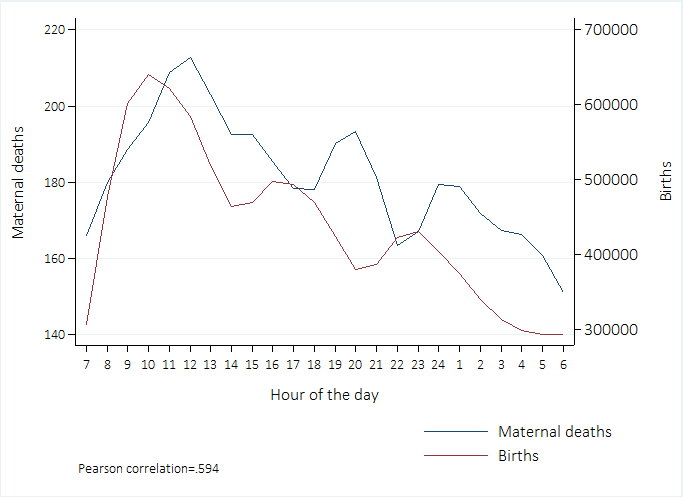 | 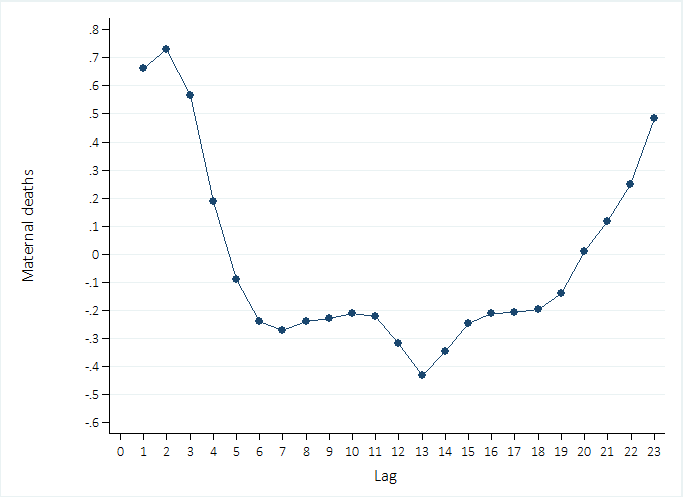 | 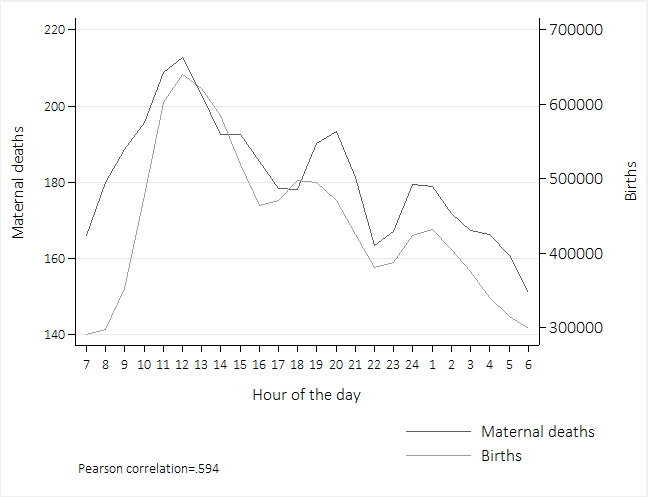 |
| **Births and maternal deaths during weekdays** | 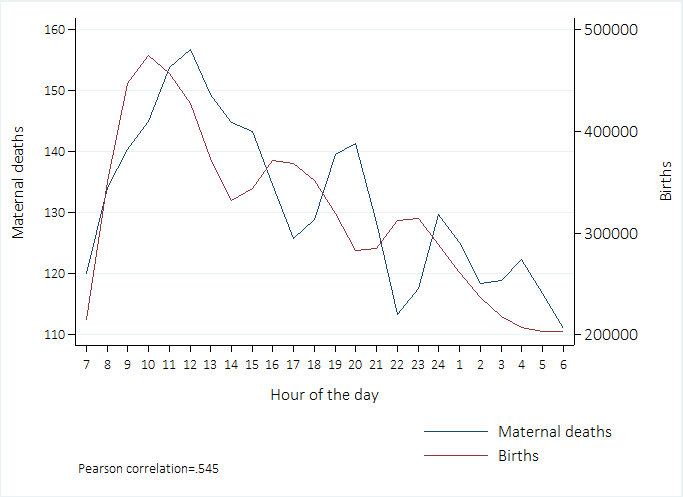 | 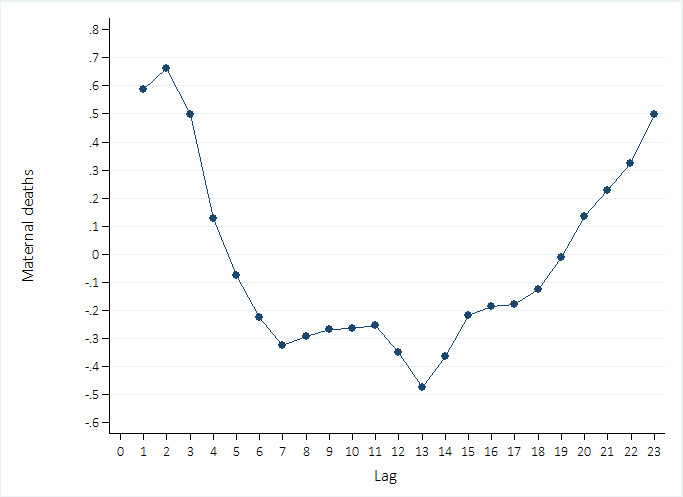 | 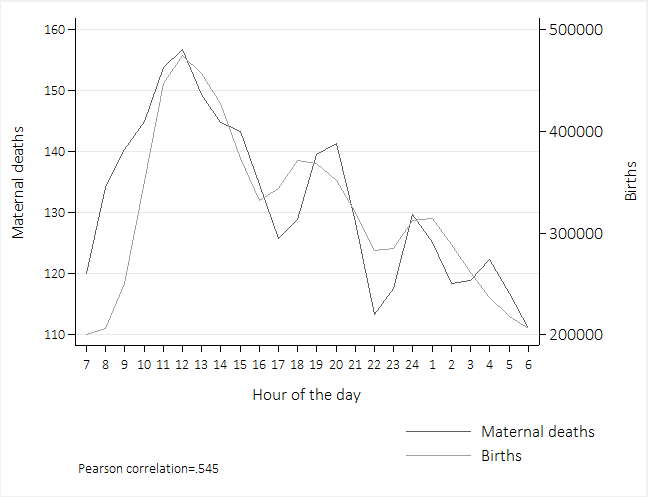 |
| **Births and maternal deaths during weekends and holidays** | 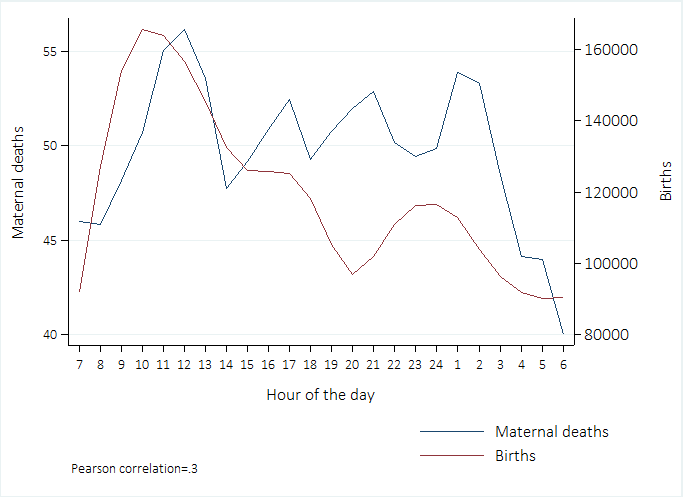 | 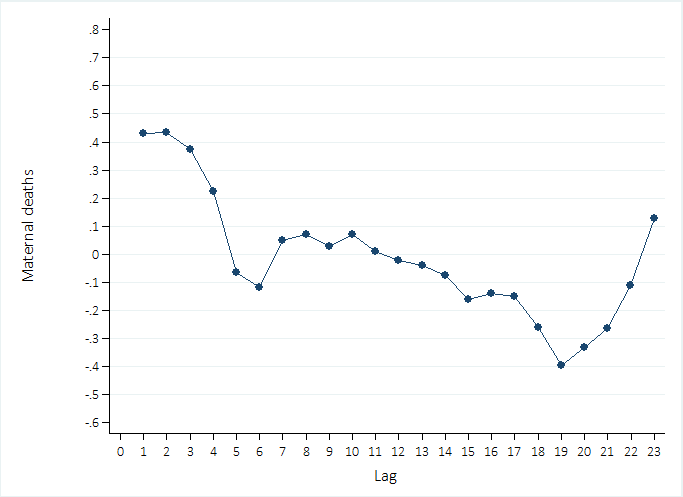 | 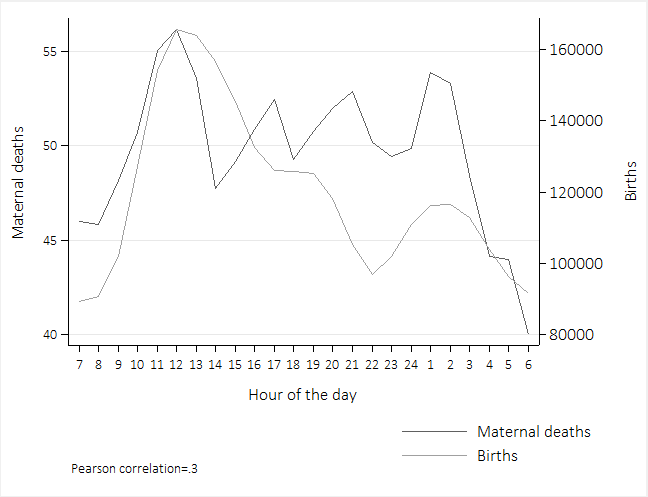 |
| **Births and maternal deaths due to abortion** | 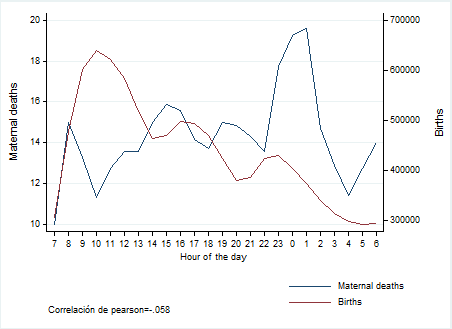 | 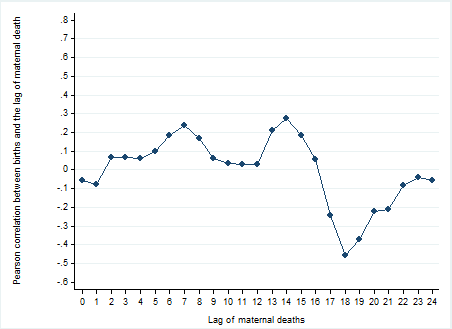 | 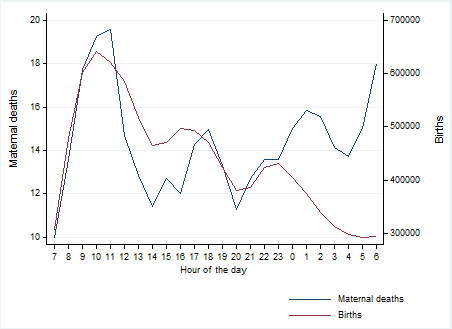 |
| **Births and maternal deaths due to hypertensive disorders of pregnancy** | 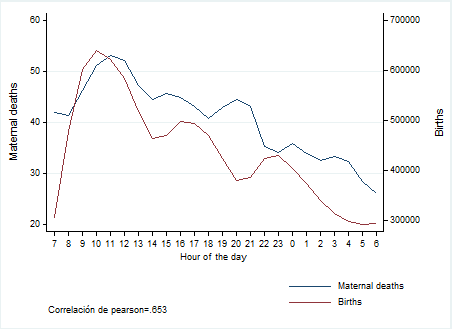 | 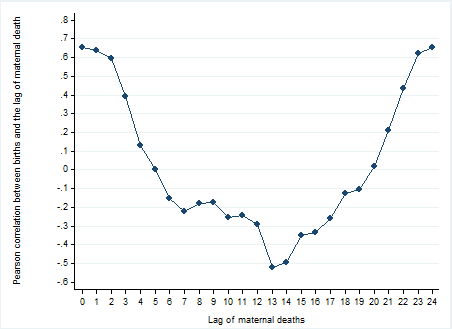 | 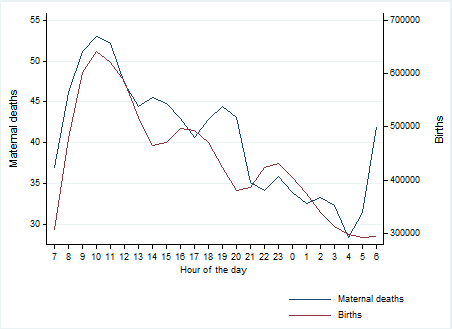 |
| **Births and maternal deaths due to obstetric hemorrhage** | 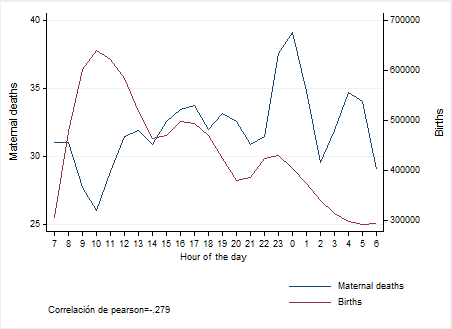 | 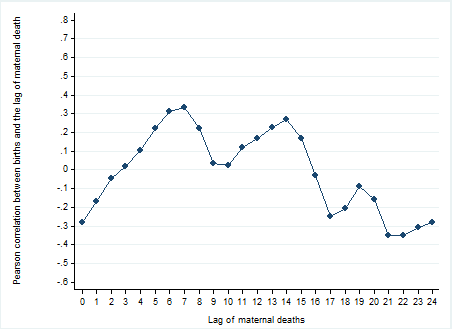 | 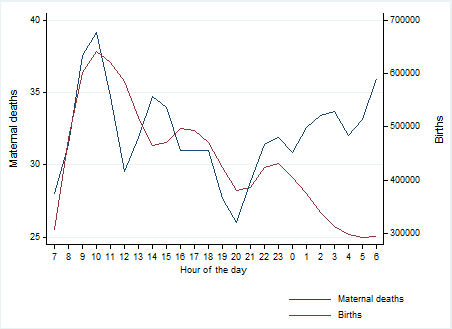 |
| **Births and maternal deaths due to indirect causes** | **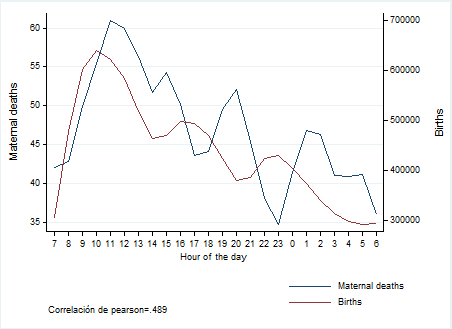** | 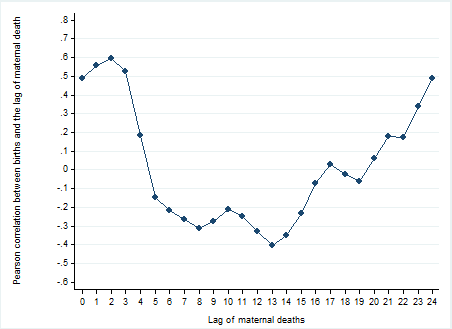 | 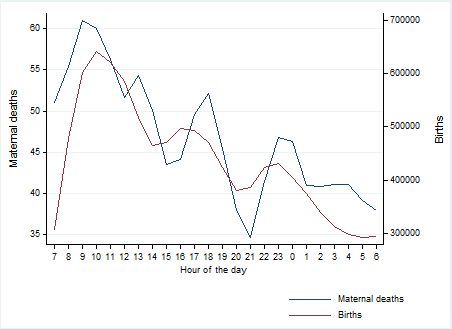 |
